# Evaluation of “Healthy Learning. Together”, an Easily Applicable Mental Health Promotion Tool for Students Aged 9 to 18 Years

**DOI:** 10.3390/ijerph16030487

**Published:** 2019-02-08

**Authors:** Susanne Schwager, Uwe Berger, Anni Glaeser, Bernhard Strauss, Katharina Wick

**Affiliations:** Institute of Psychosocial Medicine and Psychotherapy, University Hospital Jena of the Friedrich-Schiller-University Jena, 07743 Jena, Germany; uwe.berger@med.uni-jena.de (U.B.); anni.glaeser@med.uni-jena.de (A.G.); bernhard.strauss@med.uni-jena.de (B.S.); katharina.wick@med.uni-jena.de (K.W.)

**Keywords:** school health promotion, disease prevention, mental health, wellbeing, social integration, class climate, self-efficacy, program evaluation

## Abstract

Schools play an important role in adolescents’ health promotion. Due to the limited resources of teachers, there is a need for short-time interventions that can be easily implemented in a regular class without extensive training. Therefore, the tool “Healthy learning. Together.” was developed within a joint venture research project in Jena, Germany. The tool consists of a box with 60 exercises and a poster exhibition for students in 5th grade and higher. One thousand one hundred and forty four (1144) students (56% female) from nine schools were assessed at an interval of 10 weeks in a parallelized pre-post-design with class-wise assignment to intervention group (IG) and control group (CG). In the IG, regular teachers implemented the health promotion tool. Before and after the intervention social integration, class climate, self-efficacy (as primary outcomes) and mental and physical wellbeing (as secondary outcomes) were measured using standardized questionnaires. ANCOVA analysis revealed that students of the IG showed more positive changes on primary outcomes with small effect sizes. Additional implementation outcomes showed high teacher and student enthusiasm but sometimes low exposure rates. Regarding the relatively small amount of time and preparation for teachers to get noticeable effects, the introduced tool is suitable as a first step into health promotion for schools.

## 1. Introduction

Over the last decades, growing rates of mental health concerns among children and adolescents have been observed. Studies show that 10–20% of children and adolescents are suffering from severe mental health problems [1,2]. Specifically, mood disorders show a twofold increase from the age of 13–14 years to the age of 17–18 years and affect up to 11% of the population. Substance use typically occurs at the age of 15 and up to 11% of the adolescents suffer from substance use disorders [1]. Similarly, behavior disorders emerge at the age of 11 years and affect nearly one fifth of the adolescents. Moreover, about one third of adolescents suffer from anxiety disorders [1]. At the same time, physical wellbeing becomes impaired due to rising rates of obesity and physical inactivity [3,4]. Also pain or psychosomatic symptoms are a common experience among adolescents with increasing rates during this stage of life [5]. Given these alarming rates of mental and physical health problems among this young age group and the high rates of relapse and lifelong prevalence [6], the question arises what can be done to promote adolescents’ health and wellbeing (Across the sectors of educational, clinical and social psychology, terms sometimes differ. In the following, mental wellbeing and health will be used interchangeably with emotional wellbeing or health).

A promising approach to this question is to identify causes of adolescents’ impairments in their health and wellbeing. From a social perspective, a broad body of literature reveals the importance of social integration (In the literature, other terms like social inclusion, belonging, social climate or social wellbeing are applied interchangeably to refer to social integration. In the following we will use the term social integration) for health and wellbeing [7,8,9,10,11]. Social integration is characterized by frequent positive interactions with others, connectedness, and mutual appreciation [7]. It is a fundamental human need and its absence affects health and wellbeing through various direct and indirect paths [7,8,10]. A lack of social integration or even the experience of exclusion is associated with mental and physical impairments. Adolescents are confronted with significant changes in their social interactions, typically involving a detachment from parents and stronger peer influences [12]. This makes them more susceptible to the detrimental influence of social exclusion in school. Accordingly, studies show that rejection, exclusion, or bullying are common experiences in adolescence and have a strong impact on adolescents’ health and wellbeing [13,14,15,16]. Likewise, good class climate can contribute to wellbeing and academic achievement [17,18]. Given the influence of social integration on adolescents’ health, processes leading to a better social integration in school should be an essential part of health promotion programs.

Alongside social influences, psychological factors interact with health and wellbeing as well. Many theories on changing unhealthy behavior as well as promoting health behavior highlight the importance of self-efficacy, i.e., the confidence in one’s own competences and abilities [8,19,20,21]. In all of these theories, self-efficacy is seen as a core belief that has an impact on health relevant processes such as health habits and actions, motivation and perseverance, ability to recover from setbacks and relapses. The empirical evidence regarding the influence of self-efficacy on various aspects of health is extensive. Self-efficacy has been shown to increase adolescents’ physical activity [22], reduce risk behavior or drug consumption [20,21] as well as physical and psychological illness among children or students [23,24]. Furthermore, it even predicts less distress and better somatic health after trauma [25,26] and is negatively associated with anxiety and mental health problems in adolescents [27,28]. Accordingly, self-efficacy has been a key target of several health promotion programs [29,30,31,32]. 

Schools represent an ideal context for health promotion outside healthcare settings as they allow reaching a broad range of students in a cost-effective way and mitigating negative impacts of other social environments [33,34]. Health promotion programs focus on a broad variety of topics such as physical health (e.g., nutrition, physical activity), substance abuse, sexual risk behavior or emotional or social skills [35]. Most of these programs aim to improve coping skills and in this vein also self-efficacy of the students [34]. For instance, programs on substance abuse or physical activity oftentimes concentrate not only on knowledge transfer but also aim to strengthen self-efficacy [36,37,38]. Social integration has also been the target of several health promotion interventions and anti-bullying programs [39,40,41]. 

Thus in sum, social integration and class climate as an indicator of social integration, respectively, and self-efficacy are essential elements of health promotion programs. However, most of these programs are quite challenging for schools to implement. Many programs that aim to promote health by social integration for instance require specifically trained staff and/or considerable class time or project days [40,41,42,43,44]. Recently, great efforts have been made to develop whole-school programs which support schools in creating a healthy environment that promotes mental and physical wellbeing and improves the school climate [33,34,45]. These programs aspire to place social and emotional wellbeing throughout the school’s curriculum and to work collaboratively with all parts of the school community. While whole-school programs have shown to be effective in general [33,34], mixed evidence and disadvantages are reported as well [45]. Despite the potential benefits, whole-school approaches are often challenging since they require time, training and supervision or specialists like educational psychologists [33] and the flexibility and willingness of schools to change the present curriculum. 

Accordingly, school health programs oftentimes face a considerable science-to-practice-gap [35,43,46]. In Germany, only 13.7% of all schools actually implement health promotion programs and only 50% view health promotion as part of their school profile [47]. Reasons might be the extra burden of a new curriculum or health program and insufficient capacities to become acquainted with the program [35]. In their review, O’Reilly and colleagues [33] concluded that due to these implementation issues it may be more beneficial in some cases to apply smaller interventions, i.e., tools instead of large whole school programs. 

Thus, health promotion tools are required that on the one hand target self-efficacy, social integration and class climate as core correlates of health and on the other hand respect the limited resources of teachers and schools for health promotion interventions. Therefore, we developed a demand-based tool in cooperation with teachers and students that constitutes a first low-threshold step into health promotion [48,49]. The tool entitled “Healthy learning. Together.” [Gemeinsam Gesund Lernen] consists of two modules: a box with short and easy exercises and a poster exhibition (for more details see methods section as well as Schwager et al. [49]). It was developed according to the Society of Prevention Research in a three-step evaluation [48,50]. The aim of the present study was to evaluate the newly developed school-based health promotion tool “Healthy learning. Together.” regarding its efficacy, that is, its direct influence on social integration, class climate and self-efficacy as resources for wellbeing and physical health of students.

## 2. Materials and Methods 

### 2.1. Study Design 

The tool was evaluated using a parallelized pre-post-design with class-wise assignment to intervention and wait list control group. Both intervention and control group took part in a pre and post measurement with standardized questionnaires at an interval of 10 school weeks plus two weeks of vacation in the time period of September and December 2016. Additionally, we conducted semi-structured interviews (Supplementary Materials) with students and teachers after the intervention period. The study was approved by the ethics committee of the University Hospital Jena of the Friedrich-Schiller-University Jena (#4477-07/15).

### 2.2. Participants 

**Figure 1:**
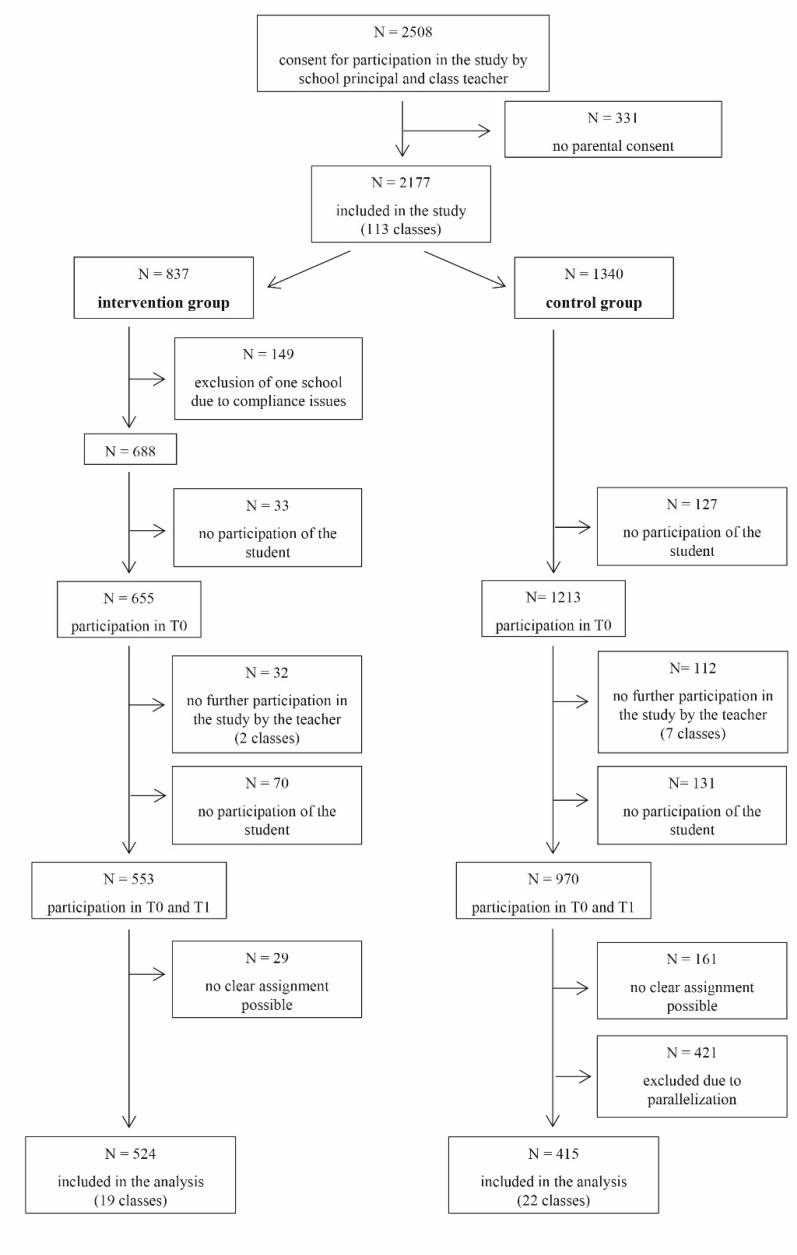
Sample flow chart for the questionnaire survey.

After presenting the tool and obtaining permission from the teaching staff, 2508 students of ten secondary German schools were asked to participate in the study (see Figure 1). We received informed consent from 2177 students and their parents. One school had to be excluded from the intervention group due to compliance issues such that they did not ensure that pre measurement took place before the exercises were conducted. At the first measurement, 160 students were not able to fill out the questionnaire due to organizational or motivational problems (e.g., students being not at school on the day of the survey because they were ill or changed school or students not willing to participate). Thus, 1868 students filled out the baseline questionnaire. At the second measurement nine teachers no longer participated in the study (Some teachers did not trust in pseudonymity as students received a personal code to match first and second measurement even though pseudonymity was guaranteed and ensured. Other teachers did not have enough time to conduct the survey due to personal illness and resulting cancellation of lessons). This resulted in a drop out of 144 students. Additionally, 201 students were not able or willing to participate at the second measurement. The assignment of 190 questionnaires to the corresponding test person was not possible so that the comparison between pre and post measurement would have been problematic. Then, we paralleled the sample of intervention and control group according to age and school type resulting in a final sample of 912 students.

In this parallelization process we aimed to reach similar age and school type distribution and randomly discarded students of the control group to align age and school type as recommended by Stuart [51]. Since there was not always a perfect matching student compared to the intervention group (e.g., student of the intervention group were on average younger) we had to discard more students of the control group. The final sample (58.5% female) included students with an age range from 10 to 17 years with an average age of *M* = 12.43 years (*SD* = 1.68). Further sociodemographic sample characteristics along with the initial outcome scores are depicted in Table 1. About one month after the questionnaire assessment, we conducted interviews with 15 teachers (13 female, all participating teachers in the intervention group) and group wise interviews with 88 students (43 female) who participated in the intervention group. Additionally we asked 40 teachers to fill out a booklet to evaluate the exercises of the tool box and received 34 answered booklets. 

### 2.3. Health Promotion Tool

In the present study we applied the tool “Health learning. Together”. This tool was developed in the course of an intense pilot study and in cooperation with students and teachers [48,49]. In a demand analysis, the structural requirements for school-based prevention tools were assessed by semi-structured interviews with 20 pedagogues from three schools in focus groups. The materials were developed and improved step by step as a result of the feedback of the pedagogues. Based on the results of the demand analysis, we developed a card set with 60 exercises for usage in school lessons and a poster exhibition with 10 posters with the title "belonging" for the school building. 

For the exercises in the card set, the challenges school professionals observe in their students in everyday school life were collected and summarized in five categories: social integration, self-esteem, emotions, life competencies and concentration. For these categories, exercises were developed that last about 10 min and are printed on index cards. Each card contains short instructions how to perform the exercise as well as a summary of the objective and symbols concerning duration, recommended age, place, and material needed. On the back of each card teachers find reflection advices and questions leading through a discussion. One example for an exercise of the category self-esteem is named “Strength bombardment” where students are instructed to compliment each other. To strengthen the social integration, exercises were selected that involve working together on several tasks, pursuing collective goals, and establishing contact between subgroups. All exercises are easy to implement by regular teachers in the course of normal lessons. Additionally, the card set includes one card per poster (for details see following paragraph) to enable the teachers to work more profound on these topics in the classroom.

Each intervention group school had access to the poster exhibition consisting of 10 posters which were set up in the school building. For the poster exhibition, 10 topics were identified as typical challenges and resources in childhood and adolescence, such as friendship, media, tolerance, anxiety, addiction, or depression. The posters depict a large attention-grabbing photograph and three facts as well as three behavioral advices regarding the depicted topic. The posters were intended to sensitize students regarding typical age-related difficulties, to encourage an exchange on the topics, and to point out further sources of information. Further details of the tool (exercises and poster) are described elsewhere [49]. 

The universal prevention tool was designed for students aged 9 to 18 years old. The tool was developed to be self-explaining. Therefore, teachers did not receive a longer period of training or supervision. The materials were tested for feasibility in these three schools by using the prevention tool in the classroom within 6 weeks. Furthermore, an accompanying process evaluation with derivation of improvements, especially from the participants’ view was conducted. Details of this feasibility study are reported elsewhere [48]. Most importantly, we derived from the data of this study, that teachers should implement 15 exercises within 10 weeks. Accordingly, teachers of the present study were instructed to implement 15 exercises.

### 2.4. Measures

#### 2.4.1. Implementation Outcomes

We assessed four different aspects of implementation according to Dane and Schneider [52]: adherence, exposure, quality of delivery and participant responsiveness. Therefore we conducted semi-structured interviews with teachers and students. These interviews were transcribed and statements were categorized according to a scheme to the four aspects of implementation and rated as positive, neutral, or negative by two independent research assistants (see Table 2). In case of disagreements, two of the authors categorized the respective statements and evaluated them as positive, neutral, or negative. To increase trustworthiness of our data we applied triangulation, i.e., we applied different sources (different teachers and students) and different researchers evaluated independently the quotes. We stopped interviewing teachers after qualitative data reached saturation. Regarding adherence, teachers were asked whether they delivered the components as prescribed in the tool box. Exposure was assessed by asking the teachers, whether they conducted the amount of exercises as suggested. Quality of delivery was examined regarding teachers enthusiasm and session effectiveness estimated by the teacher. Each new content point was counted. Participant responsiveness was derived from the student interviews. Students were interviewed in small groups of one to three students. Per student only one statement regarding the exercises of the tool box and the poster exhibition was counted. Additionally, teachers of the intervention group received an extra evaluation booklet where they indicated whether they conducted a respective exercise (exposure). Moreover, they briefly rated on a 3-point scale (low, medium, high) for each conducted exercise whether they fulfilled the aim of the exercise (adherence), the exercise’s estimated effectiveness (quality of delivery) and student participation (participant responsiveness).

#### 2.4.2. Primary and Secondary Outcomes

*Social integration*. Social integration was measured by the Social Integration Scale by Haeberlin, Moser, Bless, and Klaghofer [53], (e.g., “I get along well with my class mates” or “I feel alone in my class”) comprising four items with a four point Likert scale ranging from 0 (low social integration) to 3 (high social integration). This scale showed good internal consistency with Cronbach’s alpha of .815. 

*Class climate*. The class climate was assessed by the Linzer Fragebogen zum Schul- und Klassenklima (Linzer Questionnaire of School and Class Climate) by Eder and Mayr [54] comprising six items with a four point Likert scale ranging from 0 (low social integration) to 3 (high social integration), e.g., “We have a really good class community”. This scale showed an internal consistency with Cronbach’s alpha of .684, which is sufficient for group comparisons. 

*Self-efficacy*. To assess self-efficacy we applied the widely used German Generalized Self-Efficacy Scale [55] with ten four point Likert scale items ranging from 0 (low self-efficacy) to 3 (high self-efficacy). An example of an item would be “Thanks to my resourcefulness, I can handle unforeseen situations.” The internal consistency with Cronbach’s alpha of .852 was good. 

*Mental and physical wellbeing*. Wellbeing as secondary outcome was assessed by the mental and physical wellbeing subscales of the in Germany well-established KINDL-R Questionnaire [56]. Both scales excel in shortness and comprise four five point Likert scale items (e.g., for mental wellbeing “During the last week I felt lonely”, e.g., for physical wellbeing “During the last week I felt ill”), both ranging from 0 (low wellbeing) to 4 (high wellbeing). Regarding the mental wellbeing scale, a low internal consistency with Cronbach’s alpha of .535 was found. With respect to the physical wellbeing scale, an internal consistency with Cronbach’s alpha of .650 was reached.

### 2.5. Statistical Procedure

Data were analyzed using SPSS Statistics for Windows Version 22 (Released 2013, IBM Corp., Armonk, NY, USA). First, we examined the extent of missing data. The results of Little’s MCAR test [57] indicate that missing values were completely at random, χ^2^(143) = 136.18, *p* = .645. Considering the low percentage of missing values for all variables except for sex (sex = 11.1%, all other variables < 2.9%), we did not impute missing data. As students were potentially nested within classes and schools, we tested for the need of hierarchical analyses. However, this could be rejected as the intraclass correlation (ICC) for all dependent variables revealed no substantial differences between the various schools and classes (all *ICC*s < .019 for class and < .005 for school). Instead we performed ANCOVAs using standardized variables. Therefore, we calculated for each dependent variable a difference score between pre and post measurement with higher values indicating improved social integration, class climate, self-efficacy or wellbeing, respectively. Those difference scores served as dependent variables in the ANCOVAs. Group (control vs. intervention) served as independent variable and the pretest value (baseline) of the respective dependent variable as covariate. We applied one-tailed testing whenever testing a directional hypothesis, i.e., increasing social integration, self-efficacy, and wellbeing in the intervention group. A one-tailed test is recommended in order to increase the power for testing a directed hypothesis [58]. We calculated partial eta-square as effect sizes. According to Cohen [59] a small effect is reflected in η^2^_partial_ = .01, η^2^_partial_ = .06 represents a medium effect and a large effect can be seen by η^2^_partial_ = .14.

## 3. Results

### 3.1. Evaluation Regarding Implementation Outcomes

Implementation was evaluated according to Dane and Schneider [52] regarding adherence, exposure, quality of delivery, and participant responsiveness. Prototypical interview statements and the amount of positive, neutral or negative statements regarding these dimensions are depicted in Table 2. 

Regarding adherence, the majority (62.5%) of evaluations by the teachers was positive (12.5% neutral, 25% negative). Exposure rates were mostly (60%) negative and less often positive (40%). Most of the evaluations regarding quality of delivery were positive (69.4%) and fewer neutral (11.1%) or negative (19.4%) evaluations were registered. With respect to participant responsiveness, most of the students evaluation were positive (71.4%), followed by neutral statements (21.7%) and fewer negative evaluations (6.9%). 

Moreover, we analyzed the teacher rating on the exercises in the evaluation booklet. In sum, 513 exercises were rated by 34 teachers. Regarding exposure we found that on average teachers implemented 14.7 exercises during the 10 weeks intervention period (range: 4–60 exercises). 48.6% of the teachers implemented 15 or more exercises as suggested. Regarding self-estimated adherence, 39.7% of the teachers stated that they fulfilled the goal of the exercise to a high degree, 48.1% estimated that they moderately fulfilled the goal and only 12.1% reported low fulfillment. With respect to participant responsiveness, teachers perceived student participation in 54% of the cases as high, 37% as medium and 8.9% as low. Concerning quality of delivery, teachers estimated the effectiveness of the exercises in 45.9% of the cases as high, 40.7% as medium and 13.4% as low. 

### 3.2. Effects on Primary and Secondary Outcome Measures

First, ANCOVAs for the whole sample were conducted for the primary and secondary outcomes (see Table 3). These analyses revealed a significant group effect regarding changes in class climate, i.e., students of the intervention group showed more positive changes from pre to post measurement than students of the control group. No other group effects reached significance. Moreover, we found significant effects of the covariates (i.e., the pre measurement of the respective outcome measure) in all the conducted analyses (all *p*s < .001, all *F*s > 120.58). No interactions of the group variable and the respective covariate were significant (all *p*s > .128, all *F*s < 2.33). 

Second, we analyzed the effects for the high-adherence subgroup that followed the instructions and completed at least 15 exercises (see Table 3). These analyses showed significant group effects for the primary outcome variables class climate, social integration, and self-efficacy. Students of the intervention group showed more positive changes regarding the perceived class climate, social integration, and self-efficacy compared to the control group. For the secondary outcome measure no group effects could be observed. Again, we found significant effects of the covariates (i.e., the pre measurement of the respective outcome measure) in all the conducted analyses (all *p*s < .001, all *F*s > 85.09). No significant interactions of the group variable and the respective covariate were observed (all *p*s > .109, all *F*s < 2.580).

## 4. Discussion

In the present study, we aimed to evaluate the easily applicable health promotion tool “Healthy learning. Together.” for schools that targets social integration, class climate and self-efficacy to strengthen students’ wellbeing and health. Based on the extensive amount of research that proves the impact of social integration [7,8,9,10,11] and self-efficacy [8,17,18,19,20,21] on wellbeing, we aimed to focus on these human needs. A number of profound programs addressing this approach already exist [39,40,41,60]. However, it is oftentimes difficult for teachers to implement these programs as they are usually quite extensive and resources of teaching staff are limited [33]. Therefore, we developed an easily applicable health promotion tool consisting of an exercises box and a poster exhibition [49] and tested it in a pilot study, recently [48]. To evaluate the tools’ efficacy in the present study, we analyzed the data of 939 students of 31 classes of nine German schools who were measured twice at an interval of 10 weeks. In the intervention classes, teachers implemented the health promotion tool while students in the control group participated in education as usual. In addition to the questionnaires we evaluated the implementation by student and teacher interviews and brief evaluation questions regarding the particular exercises of the tool box. 

Regarding the implementation, we found medium to high self-estimated adherence in the rating for the exercises and mostly good evaluation statements regarding adherence in the teacher interviews. Low adherence was often reflected in teachers letting the students instruct the exercises. Exposure rates were lower than expected due to limited time of the teachers even though the exercises were constructed and tested to be very short (10 to 15 min) in our pilot study [48]. Only barely half of the teachers implemented 1.5 exercises per week. The average amount of conducted exercises was 14.7 in the course of the 10 weeks of the study. Quality of delivery as measured by the effectiveness ratings for the particular exercises was mostly medium to high. Also the interviews point to mostly positive evaluations and high enthusiasm of the teachers regarding the health promotion tool. Similarly, participant responsiveness as reflected by the student evaluations of the exercises and the poster exhibition was positive in more than two thirds of cases. Teachers also rated the student responsiveness in nine of ten cases as medium or high. 

The results of a pre-post-evaluation study revealed that students of the intervention group showed more positive changes regarding the primary outcomes social integration, class climate, and self-efficacy but only for those classes where teachers implemented at least 15 exercises a week. This matches the findings of our pilot study which also suggests implementing 1.5 exercises per week as realistic [48]. It seems that the health promotion tool is able to have an impact on the essential aspects of adolescents’ lives as social integration, class climate, and self-efficacy but only if a minimum exposure rate is given. This is a typical finding in the health promotion literature [61]. However, the presented tool does not involve long time exercises or even project days. 1.5 exercises per week means only about 10 to 30 min a week. Interestingly, even with such short exposure rates the tool is able to exert some small influence on social integration, class climate, and self-efficacy.

On the secondary outcomes physical and mental wellbeing we did not find significant effects. Even though the descriptive data points into the right direction we were not able to show an impact of our tool on these variables. This might be traced back to several causes. On the one hand, mental and physical wellbeing is subject to various influences like seasonal influences (our study took place during autumn and winter), personal experiences (e.g., academic (mis-)achievement, high work load) and other social contexts like family and peers [62]. It seems that our tool was not able to exert a significant influence above these aspects. On the other hand, we only have data on the effects of a ten-school-week-implementation of the health promotion program. Studies suggest that a longer or at best permanent implementation can increase the effects of a health promotion program [35]. From a statistical perspective, also the relatively and unexpectedly low reliabilities of the variables mental and physical health point to potentially high error variance which might also have reduced the effects. Therefore, the results on the poorly reliable wellbeing scales should not be overinterpreted [63].

Also for the primary outcomes, the effects are rather small. This is a common finding in the health promotion literature as well as in real life studies [64,65,66] and might have diverse causes. Implementation parameters like motivation, commitment, skills, or invested time have a strong impact [33,66]. Even though implementation parameters suggest rather medium to high adherence for the single exercises, we have to consider that these are self-report data which underlie biases and social desirability effects. It might be possible that external adherence ratings might have improved the accuracy of the implementation measures and would have allowed for more specific data analyses of the effects. However, to increase the external validity we aimed to test the program under conditions that are as realistic as possible. Therefore, we did not want to control teachers in their implementation and disturb their lessons too much. If we control for the amount of exercises conducted we find significant effects. Perhaps increasing the exposure rate would lead to larger effects, however, our interviews and rating data show that it was even difficult to implement 1.5 exercises per week due to the high work load of the teachers and the high amount of required educational material. This might imply the need for structural changes in the educational system (e.g., restructuring educational material, more staff) in Germany that allow teachers more time for health promotion issues. Furthermore, social integration and self-efficacy are very basic and not completely flexible constructs that are target of various internal and external influences [67,68,69]. Further influences on the feeling of social integration and self-efficacy of the students might be other contexts like family [62], other peer-groups or extracurricular activities which reduce the potential impact of a school-based tool [70]. Moreover, characteristics of the school itself and the school policy affect students’ wellbeing beyond our tool [33,69]. Accordingly, we find the largest effects for class climate which should be directly influenced by the developed health promotion tool. 

### Limitations

Some methodical limitations of the study have to be named and discussed. We had to face some drop-out especially in the control group. Due to compliance issues we also had to discard the data of an entire school within the intervention group which reduced our sample. Because of the resulting differences regarding age and school type we decided to parallel our data which reduced our sample. Additionally, we could not completely randomize the assignment of classes to intervention and control group as normal teachers had to implement the exercises and thus, we needed the agreement of the teachers to implement the tool in their lesson leading to self-selection to some degree. Similarly, we decided to do a real life study and to evaluate the tool as implemented by normal teachers. However, this reduced the possibility to completely control the implementation. In this vein, we asked teachers to only implement our tool during the time of our study and they promised to do so. However, some teachers claimed to regularly implement similar exercises even before our study which might have reduced the additional impact of our tool and might have added some error variance. Moreover, our study is not able to evaluate long term effects of the health promotion tool which should be targeted in a future study. Additionally, our study took place in central Germany and we do not have data on effects in other of parts of Germany or other countries.

## 5. Conclusions

To sum up, we find first evidence on positive effects of the developed health promotion tool on self-efficacy, class climate, and social integration. This is a promising approach since our tool represents a first step into health promotion that can be implemented by teachers in their regular school routine without an additional training or extensive manual. In our view, small interventions like our health promotion tool constitute an alternative to large-scale interventions. It has the potential to address schools that would otherwise not implement health promotion interventions at all. However, our data also shows that a minimum exposure rate of 1.5 exercises a week is necessary to produce these effects. Data on implementation shows that the tool is easy to apply and appreciated by students and teachers but that teachers sometimes still face too much work load so that they are not able to include it regularly and more frequently in addition to the normal educational material. However, given the rising rates and the strong impact of mental health problems among adolescents [1,2] resources should be pooled to face this important issue.

## Figures and Tables

**Table 1:** Sample characteristics and baseline scores.

Variable	Intervention Group (N = 524)	Control Group (N = 415)	Group Difference
**Sociodemographic variables (T1)**			
Age (years), *M* (*SD*)	12.52 (1.67)	12.60 (2.01)	*t*(936) = 0.67, *p* = .503
Sex (female)	57.9%	58.5%	*t*(833) = 0.17, *p* = .862
School type *	Secondary: 13.5% Comprehensive: 55.3% Grammar: 31.1%	Secondary: 17.1% Comprehensive: 53.3% Grammar: 29.6%	*χ*^2^(2) = 2.29, *p* = .318
**Baseline scores**			
Class climate, *M* (*SD*)	2.03 (0.53)	2.03 (0.51)	*t*(929) = 0.18, *p* = .855
Social integration, *M* (*SD*)	2.42 (0.57)	2.44 (0.55)	*t*(931) = 0.39, *p* = .700
Self-efficacy, *M* (*SD*)	1.90 (0.45)	1.91 (0.45)	*t*(926) = 0.32, *p* = .750
Mental wellbeing, *M* (*SD*)	3.08 (0.60)	3.09 (0.59)	*t*(937) = 0.22, *p* = .830
Physical wellbeing, *M* (*SD*)	2.68 (0.70)	2.66 (0.71)	*t*(937) = −0.57, *p* = .572

Note: N = number of students, T1 = first measurement, *M* = mean, *SD* = standard deviation. * Secondary schools [Regelschulen] offer a diploma either after 9th or 10th grade and qualify for vocational training. Grammar schools [Gymnasien] end with a high school diploma after 12th grade and qualify for university. In comprehensive schools [Gemeinschaftsschulen] all students are taught together until 8th grade (separation usually after 4th grade) and then decide to either finish after 9th, 10th or 12th grade.

**Table 2:** Evaluation of implementation outcomes. Number of positive, neutral, and negative evaluation of teacher and student interviews with exemplary statements.

Aspect of Implementation	Source	*n* Positive Evaluations	*n* Neutral Evaluations	*n* Negative Evaluations	Example Positive Evaluation	Example Neutral Evaluation	Example Negative Evaluation
adherence	teacher interviews	10	2	4	“It was very clear to me (…) and the tool was easy to use.”	“It was difficult to find the right time for the poster exhibition, so I asked students to visit it on their own.”	“I did not use every exercise exactly as instructed as the students were a little too tired for them.”
exposure	teacher interviews	4	0	6	“I implemented many games, especially during the first week and until the holidays.”	Not applicable	“Well, I did not manage to do everything (every exercise), because somehow something different always comes up in school.”
quality of delivery	teacher interviews	25	4	7	“(I liked) that it had different exercises and that the students actually slowly opened up.”	“Well a few (exercises) were questionable, where I needed more space in my class room. And I have a room, where the tables are fixed, so I was limited regarding the exercises from the beginning.”	“I think for the teenagers it was really cool, but for the younger ones it (the poster exhibition) was a bit scary sometimes.”
participant responsive-ness	student interviews	145	44	14	“Yeah, I mean, that kind of project brought the class closer together somehow.”	“Some (exercises) were not bad, but I think they did not do that much for us now.”	“Many classmates did not take it seriously.”

**Table 3:** Statistical values for the ANCOVAs of the primary and secondary outcome measures.

Outcome Measure	Mean Change Score Control Group (*SD* in Parentheses)	Mean Change Score Intervention Group (*SD* in Parentheses)	*F*-Value	*df*	*p*-Value (One-Tailed)	Effect Size *η*^2^_partial_
Results for the whole sample						
**Primary outcomes**						
Class climate	−0.056 (0.423)	−0.002 (0.422)	3.857	1, 920	.025 *	.004
Social integration	−0.014 (0.617)	0.033 (0.600)	0.936	1, 849	.167	.001
Self-efficacy	−0.003 (0.455)	0.042 (0.538)	1.520	1, 520	.109	.002
**Secondary outcomes**						
Physical wellbeing	−0.133 (0.721)	−0.097 (0.746)	1.426	1, 934	.117	.002
Mental wellbeing	−0.006 (0.530)	−0.008 (0.557)	0.000	1, 933	.495	.000
Results for the sub-sample with a minimum of 15 exercises.						
**Primary outcomes**						
Class climate	−0.056 (0.423)	0.026 (0.419)	4.702	1, 598	.016 *	.008
Social integration	−0.014 (0.617)	0.061 (0.609)	3.626	1, 547	.029 *	.007
Self-efficacy	−0.003 (0.455)	0.071 (0.502)	2.906	1, 558	.045 *	.005
**Secondary outcomes**						
Physical wellbeing	−0.133 (0.721)	−0.028 (0.725)	2.256	1, 606	.067	.004
Mental wellbeing	−0.006 (0.530)	.051 (0.561)	2.592	1, 607	.054	0.004

* *p* < .05, ** *p* < .01, *** *p* < .001, *SD* = standard deviation, *df* = degrees of freedom (numerator, denominator).

## Supplementary Materials

The following are available online at http://www.mdpi.com/1660-4601/16/3/487/s1, Interview Guidelines.
