# Supplementary material for: Evaluation of “Healthy Learning. Together”, an Easily Applicable Mental Health Promotion Tool for Students Aged 9 to 18 Years
Source: Int J Environ Res Public Health. 2019 Feb 8;16(3):487. doi: 10.3390/ijerph16030487 (PMC6388215; doi:10.3390/ijerph16030487)
Supplement: Supplementary file 1 [file ijerph-16-00487-s001.pdf]

Interview guidelines

<u>teacher interview (adherence, exposure, quality of delivery)</u>

Exercises

How do you evaluate the toolbox and its structure?

How do you evaluate the practicability of the box?

How do you evaluate the comprehensibility of the box?

How well could you implement the instructions?

Did you deliver the exercises as prescribed in the instructions? What difficulties did you have?

Did the exercises showed effects in your class? How do you evaluate them?

Could all students be involved?

How did the students like the exercises?

Was it possible to integrate the exercises into regular lessons?

How many exercises did you apply?

Would you continue to use the toolbox? If not, what are obstacles / barriers?

Which problems occurred?

What else seems to be important to know?

Poster

How did you like the poster exhibition? (content, representation,…)

Did you visit the exhibition with your class? What do you think, did the posters reach/affect the students? (other colleagues, parents,…)

Did you include the posters in your lessons? If so, how well did that work?

Could you imagine setting up the poster exhibition again independently of the project?

What else seems to be important to know?

student interview (participant responsiveness)

Exercises

How did you like the exercises?

Were the exercises easy to understand and to apply?

Did the exercises showed effects in your class?

How many exercises a week would you like to do?

Would you like to do more exercises out of the box?

What else seems to be important to know? (improvements, wishes, difficulties)

Poster

Did you visit the poster exhibition? How long/how often did you visit the exhibition?

How did you like the poster exhibition? (content, representation,…)

Did you get new information? What did you learn? What do you remember?

Did you work with the posters in class? If so, how well did that work?

Did you talk with someone else about the project?
